# Self-reported narrative identity in Norway: psychometric evaluation of the Awareness of Narrative Identity Questionnaire and the Narrative Identity Self-Evaluation scale with focus on personality pathology

**DOI:** 10.3389/fpsyg.2026.1834608

**Published:** 2026-06-15

**Authors:** Jens C. Thimm, Anna Dorthea Jonsson Rønning, Inga Mariel Kildedam Maurstad, Jaran Svare Lilleland, Ingvild Kalsnes Jørstad Aurebekk, Benjamin Hummelen, Majse Lind

**Affiliations:** 1 Department of Psychology, UiT The Arctic University of Norway, Tromsø, Norway; 2 Department of Child and Adolescent Psychiatry, Østfold Hospital Trust, Fredrikstad, Norway; 3 Division of Mental Health and Addiction, Oslo University Hospital, Oslo, Norway; 4 Institute for Communication and Culture, Aalborg University, Aalborg, Denmark

**Keywords:** ANIQ, narrative identity, NISE, pathological personality traits, personality functioning

## Abstract

**Background:**

Narrative identity has traditionally been assessed with rater-based codings of life event narratives. Recently, the Awareness of Narrative Identity Questionnaire (ANIQ) and the Narrative Identity Self-Evaluation (NISE) were developed to assess central features of narrative identity via self-report. The goal of the study was to examine the internal consistency, structural validity, and criterion-related validity of the Norwegian ANIQ and NISE. Because prior work has specifically linked narrative identity disturbances to personality pathology, criterion validity was investigated in relation to personality functioning and pathological personality traits.

**Methods:**

An adult sample (*N* = 330, *M*_*age*_ = 35.0 years, *SD*_*age*_ = 14.2 years, 78.5% female) completed the ANIQ, NISE, Level of Personality Functioning Scale-Brief Form 2.0 (LPFS-BF 2.0), and Modified Personality Inventory for DSM-5—Brief Form Plus (PID5BF+M).

**Results:**

The results showed that the Norwegian ANIQ and NISE had high internal consistency. While confirmatory factor analyses yielded mixed results for both instruments, subsequent exploratory factor analyses supported the instruments' proposed structures. NISE narrative disturbances showed strong associations with impaired personality functioning and pathological personality traits.

**Conclusion:**

It is concluded that the Norwegian ANIQ and NISE show satisfactory reliability, structural validity, and theoretically coherent links to personality functioning and maladaptive personality traits, underscoring the relevance of narrative identity, especially narrative disturbances, for dimensional models of personality disorders.

## Introduction

Identity is one of the most central psychological constructs, but also one of the most complex (Kaufman et al., 2015; Lind et al., 2022; McAdams, 2013). According to Erikson (1968), identity is defined by a sense of self-continuity across situations (self-sameness) and the experience of a unique self that is separate from others and that gives a sense of wellbeing, purpose, and meaning. Building on Erikson's (1968) theory, McAdams (2001) proposed that identity can be understood as an evolving, dynamic, and culturally shaped story that individuals create about themselves based on how the personal past is interpreted, the present is perceived, and the future is envisioned. This life story, or narrative identity, provides the individual with a sense of unity and purpose in life (McAdams and McLean, 2013). Narrative identity is related to other aspects of personality, such as personality traits (e.g., the Big Five) and characteristic adaptations (e.g., motives) but constitutes a separate domain of personality (Adler and Clark, 2019; McAdams and Pals, 2006). McLean et al. (2020) identified three overarching domains that cover the most essential characteristics of narrative identity: autobiographical reasoning, motivational and affective themes, and structural aspects. Autobiographical reasoning refers to reflecting on and making meaning of the life story, motivational and affective themes refer to the affective tone and motivational content of the story (e.g., agency, communion), and structural aspects refer to more formal characteristics of the narrative, such as contextual and temporal coherence (McLean et al., 2020).

Oral or written accounts of a person's life story, chapters, or salient episodes (e.g., high points, low points, or turning points) are the primary source for the assessment of narrative identity (Adler et al., 2017; Dunlop, 2021; McAdams, 2018). These recounts are rated by trained coders for the thematic, reflective, and structural features of interest (Adler et al., 2017). However, alternative methods can complement and broaden the assessment and study of narrative identity (Dunlop, 2021). For instance, self-report instruments can provide direct insight into an individual's perception and understanding of their life story that is independent from the interpretation of external raters (Dunlop, 2021; Holm and Thomsen, 2018). In addition, these measures are less time-consuming to administer and to score than coding-based methods (Holm and Thomsen, 2018). A variety of self-report inventories is available that is relevant to the study of narrative identity, some of which were developed in the field of autobiographic memory (for an overview, see Dunlop, 2021). The Awareness of Narrative Identity Questionnaire (ANIQ; Hallford and Mellor, 2017) and the Narrative Identity Self-Evaluation (NISE; Lind et al., 2025) are two measures that have emerged from the narrative identity literature and have shown promising preliminary psychometric properties.

The ANIQ was developed to assess the degree to which individuals draw on personal memories to create their identity and sense of self and experience their life story as coherent (Hallford and Mellor, 2017). Based on the work of Habermas and Bluck (2000), the ANIQ distinguishes between three forms of narrative coherence that are consecutively developed in adolescence and increasingly complex: temporal, causal, and thematic coherence. Temporal coherence refers to the ability to situate life events in a clear and orderly sequence. Causal coherence reflects the extent to which life events are experienced as meaningfully connected with each other. Thematic coherence is related to the perception of overarching patterns and themes related to the self. In addition, an awareness subscale was added based on the assumption that awareness of having a life story brings cohesion and meaning to life events, regardless of what those stories contain (Hallford and Mellor, 2017). In the development of the ANIQ, an initial pool of 48 items was reduced to 20 items through iterative exploratory factor analyses (EFAs), with each subscale represented by five positively worded items (Hallford and Mellor, 2017). A confirmatory factor analysis (CFA) supported the proposed four-factor structure of the ANIQ in a separate sample. In the two samples, the internal consistency of the subscales was high (Cronbach's alpha >0.85). The 1-week test-retest reliability exceeded 0.72 for all ANIQ scales. In both samples, it was found that the subscales were strongly intercorrelated, with correlation coefficients ranging from 0.45 (awareness with temporal coherence in sample 1) to 0.79 (causal coherence with thematic coherence in sample 2). Moderate to high correlations were found with measures of wellbeing, identity reminiscence, and thinking and talking about one's life, while the associations with psychological distress were overall weak and inconsistent (Hallford and Mellor, 2017). However, subsequent studies reported small- to medium-sized negative relationships of ANIQ scales with symptoms of depression and anxiety (Dierdorp et al., 2023; Hallford et al., 2021) and symptoms of borderline personality disorder (BPD) in adolescents (Balzen et al., 2024). Finally, significant moderate associations were found between the ANIQ scales and rater-coded coherence of turning point memories (Hallford and Mellor, 2017). Gehrt et al. (2024) reported small to medium-sized correlations of the ANIQ scales with rater-coded temporal coherence of life stories but weak and mostly nonsignificant relationships with rater-coded global coherence, motivational and affective themes, and autobiographical reasoning. Studies of the Dutch (Dierdorp et al., 2023) and Turkish (Sevim and Otrar, 2021) translations of the ANIQ have shown psychometric properties similar to the original English ANIQ, while an investigation into the Farsi version concluded with a three-factor structure of the inventory, in which the causal and the thematic coherence scales formed one factor (Mazaheri-Nejad-Fard et al., 2025). In an early adolescent sample, another study on the ANIQ found support for the proposed four-factor structure of the inventory and small to moderate negative correlations with BPD features and identity diffusion (Balzen et al., 2024).

The content of the ANIQ overlaps with the framework of narrative variables proposed by McLean et al. (2020). That is, awareness and thematic coherence can be considered autobiographical reasoning variables, and the temporal and causal coherence scales fall within the structure domain. However, the motivational and affective domain is not covered by the ANIQ (Lind et al., 2025).

The Narrative Identity Self-Evaluation (NISE; Lind et al., 2025) was developed to cover all three major dimensions of narrative identity identified by McLean et al. (2020). In addition, a narrative disturbances scale was included to assess maladaptive narrative identity, complementing the assessment of the normal-range narrative identity dimensions of the McLean et al. (2020) framework. Narrative disturbances such as low autobiographical reasoning, negative emotional tone, low agency and communion, and low coherence have been repeatedly observed across different mental disorders (Cowan and Lind, 2024), especially in personality disorders (see Lind et al., 2020; for a review). With the introduction of the alternative model for personality disorders (PDs) in DSM-5 (DSM-5 AMPD; American Psychiatric Association, 2013) and the eleventh edition of the International Classification of Diseases [ICD-11; World Health Organization (WHO), 2019/2021], the assessment of identity problems has received a central role in the diagnosis of personality pathology. It has been suggested that narrative identity is highly relevant to these new models of PDs (Adler and Clark, 2019; Lind, 2021) or may even be a driver or nexus of the disorder (Sharp, 2022; Sharp and Wall, 2021). In brief, in the DSM-5 AMPD and ICD-11, PDs are defined by impaired personality functioning with respect to the self (including identity and self-direction) and interpersonal relationships (including empathy and intimacy) and further characterized by pathological personality traits (optional in ICD-11). Some authors have argued that narrative identity is particularly well-suited to capturing the various elements of self-functioning that are delineated in these models (Lind et al., 2022; Sharp, 2022; Sharp and Wall, 2021).

The NISE was constructed in a similar way to the ANIQ, starting with a list of 88 potential items from which 61 items were selected and reduced to 20 items in a series of EFAs and evaluations of item redundancy and readability (Lind et al., 2025). The resulting factors were labeled autobiographical reasoning, desire for structure, positive themes, and narrative disturbances and encompassed five items each. The autobiographical reasoning and desire for structure factors were highly correlated (0.58), while the remaining factor intercorrelations were below 0.25. A CFA conducted on a second sample showed an acceptable model fit for the four-factor model for participants with and without self-reported psychiatric diagnoses. Further, the associations of the NISE scales with measures of autobiographical memory, personality traits, self-concept clarity, and wellbeing were examined, including the ANIQ and the Levels of Personality Functioning Scale-BF 2.0 (LPFS-BF 2.0; Weekers et al., 2019), which assesses self and interpersonal functioning consistent with the DSM-5 AMPD. Overall, the NISE scales showed moderate to large correlations with the ANIQ scales. In both samples, the highest correlations were found between NISE autobiographical reasoning and ANIQ awareness (*r* = 0.52) and ANIQ thematic coherence (*r* = 0.50). NISE narrative disturbances exhibited small to moderate negative associations with all ANIQ scales. NISE narrative disturbances was strongly correlated with impaired self and interpersonal functioning, with effect sizes (*r*) ranging from 0.53 to 0.58. Lind et al. (2025) also examined the relationships between the NISE scales and rater-coded autobiographical reasoning, motivational and affective themes, and structural elements of turning point narratives. NISE autobiographical reasoning showed small to moderate correlations with rater-coded structural elements, NISE positive themes was moderately correlated with rater-coded autobiographical reasoning and motivational and affective themes, and NISE narrative disturbances showed small to moderate negative correlations with all three rater-coded dimensions of narrative identity. NISE desire for structure was not significantly related to the rater-coded variables.

Taken together, the ANIQ and the NISE have demonstrated promising psychometric properties as self-report instruments for assessing core aspects of narrative identity; however, the psychometric qualities of these inventories have not yet been investigated in Norway. Thus, the purpose of the present investigation was to examine the internal consistency, structural validity, and criterion validity of the Norwegian ANIQ and NISE. Given the centrality of identity impairment for personality pathology in the DSM-5 AMPD and the ICD-11, the present study examined the instruments' criterion validity in relation to personality functioning and pathological trait domains. Based on prior research on the ANIQ and NISE reviewed above, it was expected that the Norwegian versions of the instruments' scales would show adequate internal consistencies and that support for a four-factor structure for both inventories would be found. It was further hypothesized that the Norwegian ANIQ and NISE scales would be mostly moderately correlated but that strong associations would be found between the conceptually related NISE autobiographical reasoning scale and the ANIQ awareness and ANIQ thematic coherence scales. In addition, negative associations between the NISE narrative disturbances scale and the ANIQ scales were expected. Finally, prior research suggests that narrative disturbances align closely with the core impairments of personality pathology in the DSM-5 AMPD and ICD-11. Accordingly, it was hypothesized that NISE narrative disturbances would show higher correlations with impaired personality functioning and maladaptive personality traits than would the normal-range NISE scales as well as the ANIQ scales. It was further expected that NISE narrative disturbances would be more strongly related to impaired personality functioning than maladaptive personality traits.

## Methods

### Participants

We aimed at recruiting at least 300 participants to ensure an adequate sample size for factor analyses of the ANIQ and NISE items (see MacCallum et al., 1999). In total, 388 participants were recruited by sharing a link to a digital survey via social media, email, and posters on the university campus, across two rounds of data collection. Participants were informed about the aim and design of the study, advantages and disadvantages of participation, anonymous and voluntary participation, and the researchers' contact information. Upon completion of the survey, participants were given the opportunity to enter a raffle for four gift cards ranging from 300 to 700 Norwegian kroner (approximately 25–58 Euro). The project was evaluated and approved by the Department of Psychology's internal research ethics committee (IPS-REC; ref. nos. 10-2024, 02-2025). The Norwegian Agency for Shared Services in Education and Research's data protection services (SIKT) was notified about the processing of personal information in the raffle of the gift cards (ref. nos. 560052, 946902).

Based on two attention check questions in the survey, which instructed the participants to select a specified response option, 58 participants were excluded from the investigation, resulting in a final sample of 330 participants with a mean age of 35.0 years (*SD* = 14.2 years, range = 16 to 68 years; one implausibly high age response was recorded as missing). Most participants identified themselves as female (78.5%), 21.2% as male, and 0.3% as “other.”

## Measures

### Awareness of Narrative Identity Questionnaire Awareness of Narrative Identity Questionnaire (ANIQ)

The ANIQ (Hallford and Mellor, 2017) is a 20-item self-report inventory that comprises the four subscales awareness, temporal coherence, causal coherence, and thematic coherence as described above, containing five items each (for example items, see Table 1). Items are grouped by subscale and rated on an 11-point scale ranging from 0 to 10 with the endpoints defined as “completely disagree” and “completely agree.” The ANIQ was translated from English to Norwegian by Ingvild Kalsnes Jørstad Aurebekk and Benjamin Hummelen. It was back-translated to English by a native English internal medicine specialist with experience from mental health care, who is fluent in Norwegian. The final version was approved by David Hallford, one of the authors of the ANIQ. The Norwegian translation of the ANIQ is available in the Supplementary material.

**Table 1:** Overview of the ANIQ and NISE scales with example items.

Scale	Example item
ANIQ	
Awareness	I use my stories about my life to work out the kind of person I am.
Temporal coherence	I can put the events of my life in order of when they occurred.
Causal coherence	I understand how my life experiences are associated with one another.
Thematic coherence	I can perceive common themes about who I am across memories of my life
NISE	
Autobiographical reasoning	Thinking about my life story helps me know who I am as a person.
Desire for structure	It matters to me to have a coherent life story.
Positive themes	Overall, I would consider my life story to be more positive than negative.
Narrative disturbances	My life story feels like a puzzle and the pieces don't fit together.

### Narrative Identity Self-Evaluation scale (NISE)

The NISE (Lind et al., 2025) is a self-report inventory designed to assess the main domains of narrative identity suggested by McLean et al. (2020) with the three subscales autobiographical reasoning, desire for structure, and positive themes and an additional subscale assessing narrative disturbances (for example items, see Table 1). The five items of each subscale are presented in groups in the inventory. Responses to the items are given on a 7-point scale ranging from 1 (*strongly disagree*) to 7 (*strongly agree*).

As the NISE was developed concurrently in Danish and English and given the high linguistic similarity between Danish and Norwegian, the questionnaire was translated from Danish into Norwegian by Ingvild Kalsnes Jørstad Aurebekk and Benjamin Hummelen. Subsequently, it was back-translated to English by the same clinician who back-translated the ANIQ. The final English translation was compared with the Danish version and approved by Majse Lind, the first author of NISE. The Norwegian version of the NISE is included in the Supplementary material.

### Level of Personality Functioning Scale-Brief Form, version 2.0 (LPFS-BF 2.0)

The LPFS-BF 2.0 (Weekers et al., 2019) is a 12-item self-report inventory designed to assess personality functioning consistent with the DSM-5 AMPD. Each item corresponds to one of the twelve subdomains of the LPFS, which implies that the first six items are dedicated to assessing self-functioning, while the last six items assess interpersonal functioning. The items are answered on a scale from 0 (*very false or often false*) to 3 (*very true or often* true). Weekers et al. (2023) suggested cutoff values of 26 for mild/subclinical dysfunction and 31, 36, and 41 for moderate, severe and extreme dysfunction, respectively. The Norwegian translation of the instrument has shown adequate reliability and validity in a clinical sample (Paap et al., 2024).

### Modified Personality Inventory for DSM-5—Brief Form Plus (PID5BF+M)

The PID5BF+M (Bach et al., 2020) is based on the Personality Inventory for DSM-5 (PID-5; Krueger et al., 2012) and the Personality Inventory for DSM-5 Brief Form Plus (PID5BF+; Kerber et al., 2022) and is designed to assess the pathological personality trait domains of the DSM-5 AMPD and ICD-11, i.e., negative affectivity (emotional lability, anxiety, and separation insecurity), detachment (withdrawal, anhedonia, and intimacy avoidance), antagonism (manipulativeness, deceitfulness, and grandiosity), disinhibition (irresponsibility, impulsivity, and distractibility), psychoticism (unusual beliefs and experiences, eccentricity, and perceptual dysregulation), and anankastia (perfectionism, rigidity, and orderliness). The PID5BF+M includes 36 statements that are rated on a four-point Likert scale from 0 (*very false or often false*) to 3 (*very true or often true*) and has been validated in Norway (Bach et al., 2020).

## Analyses

Descriptive statistics for the study scales were calculated, including Cronbach's α and McDonald's ω to examine internal consistency. There were no missing data points in the study measures to be handled. The associations of the ANIQ and the NISE scales with age and gender were analyzed using Pearson correlations and independent *t*-tests, respectively. The internal structure of the ANIQ and NISE was examined using confirmatory factor analysis (CFA). Given the relatively high number of response categories (11 in the ANIQ and seven in the NISE), robust maximum likelihood estimation was used rather than a categorical estimator. Conventional model fit indices and threshold values were employed to evaluate overall model fit, i.e., Comparative Fit Index (*CFI*) and Tucker-Lewis index (*TLI*) ≥ 0.90, and Root Mean Square Error of Approximation (*RMSEA*) ≤ 0.10, for acceptable model fit and *CFI/TLI* ≥ 0.95, and *RMSEA* ≤ 0.06, for good model fit. The structure of the ANIQ and NISE items was further examined with minimum residual EFAs. The Empirical Kaiser Criterion (EKC; Braeken and van Assen, 2017) and parallel analysis with 1,000 simulated random datasets and the average EFA-based eigenvalues were used to estimate the number of factors. Factors were then obliquely rotated using oblimin. Finally, the relationships between the ANIQ, NISE, LPFS-BF 2.0, and PID5BF+M scales were analyzed using Pearson correlations. Correlation coefficients of 0.10, 0.30, and 0.50 were interpreted as indicating small, medium, and large effect sizes, respectively (Cohen, 1992). The data were analyzed in R (version 4.5.1; R Core Team, 2025) and with the packages misty (version 0.7.3; Yanagida, 2025), MBESS (version 4.9.41; Kelley, 2025), lavaan (version 0.6-19; Rosseel, 2012), EFAtools (version 0.6.0; Steiner and Grieder, 2020), and psych (version 2.5.6; Revelle, 2025).

## Results

### Descriptives

Descriptive statistics for the study measures are shown in Table 2. Based on suggested cutoff values for the LPFS-BF 2.0 (Weekers et al., 2023), most participants (78.7%) showed no impairment in personality functioning, 13.6% mild impairment, 4.5% moderate impairment, and 2.1% severe impairment. Internal consistency coefficients for the ANIQ scales ranged from 0.87 (awareness) to 0.96 (temporal coherence) for both Cronbach's α and McDonald's ω. For the NISE scales, Cronbach's α ranged from 0.78 (positive themes) to 0.85 (autobiographical reasoning) and McDonald's ω ranged from 0.80 (positive themes and narrative disturbances) to 0.86 (autobiographical reasoning). Thus, the hypothesis of adequate internal consistencies of the ANIQ and NISE scales was supported. NISE autobiographical reasoning and LPFS-BF 2.0 interpersonal functioning showed elevated values for skewness and kurtosis.

**Table 2:** Descriptive statistics and internal consistency of study measures.

Scale	*M*	*SD*	Skewness	Kurtosis	α	ω
ANIQ						
Awareness	6.62	1.69	−0.46	0.92	0.87	0.87
Temporal coherence	6.16	2.41	−0.25	−0.79	0.96	0.96
Causal coherence	6.44	1.94	−0.45	0.11	0.92	0.92
Thematic coherence	6.66	1.89	−0.67	0.82	0.94	0.94
NISE						
Autobiographical reasoning	5.73	0.93	−1.32	2.71	0.85	0.86
Desire for structure	4.72	0.99	−0.26	−0.20	0.81	0.82
Positive themes	4.95	1.07	−0.70	0.15	0.78	0.80
Narrative disturbances	2.51	1.08	0.86	0.23	0.79	0.80
LPFS-BF 2.0						
Total score	8.64	5.98	0.85	0.40	0.85	0.85
Self-functioning	5.53	3.91	0.65	−0.17	0.83	0.83
Interpersonal functioning	3.11	2.77	1.31	1.76	0.69	0.69
PID5BF+M						
Negative affectivity	1.11	0.67	0.09	−0.95	0.80	0.82
Detachment	0.63	0.53	0.75	−0.03	0.76	0.76
Antagonism	0.62	0.50	0.97	0.91	0.75	0.76
Disinhibition	0.80	0.57	0.54	−0.24	0.77	0.80
Psychoticism	0.71	0.57	0.76	0.20	0.77	0.78
Anankastia	0.87	0.67	0.62	−0.55	0.85	0.85

ANIQ, Awareness of Narrative Identity Questionnaire; LPFS-BF 2.0, Level of Personality Functioning Scale-Brief Form version 2.0; NISE, Narrative Identity Self-Evaluation; PID5BF+M, Personality Inventory for DSM-5- Brief Form Plus Modified.

The means of the four ANIQ scales did not differ between men and women at a statistically significant level (*p* < 0.05). The relationships between age and the ANIQ were neither statistically significant (*p* > 0.05 for all correlations). For the NISE, however, several significant associations emerged. First, women had significantly higher mean scores on autobiographical reasoning than men [*M* = 5.83, *SD* = 0.83 vs. *M* = 5.39, *SD* = 1.18; *t*_(327)_ = 3.54, *p* < 0.001, *d* = 0.43]. Women also had higher mean scores on desire for structure [*M* = 4.78, *SD* = 0.98 vs. *M* = 4.51, *SD* = 1.01; *t*_(327)_ = 2.03, *p* < 0.05, *d* = 0.27] than men. Moreover, age was negatively correlated with NISE autobiographical reasoning (*r* = −0.17, *p* < 0.01) and narrative disturbances (*r* = −0.16, *p* < 0.01).

### Factor structure of the ANIQ

In the CFA of the ANIQ items, the *CFI* (0.97) and the *TLI* (0.97) indicated a good model fit, whereas the *RMSEA* (0.12) suggested a poor model fit. In the subsequent EFA, parallel analysis indicated extracting four factors. The first five observed eigenvalues were 9.888, 2.438, 0.837, 0.477, and 0.045 compared with the mean eigenvalues of 0.559, 0.397, 0.335, 0.279, and 0.229 from random data. In contrast, the EKC proposed three factors. Given these ambiguous indications, two EFAs with oblimin rotation were conducted to examine both the three- and four-factor solutions.

Factor loadings and factor correlations are presented in Table 3. In the three-factor solution, the items of the awareness and temporal coherence scales defined separate factors, while the third factor was defined by the items of the causal and thematic coherence scales. One item from the awareness scale had a substantial secondary loading (0.31) on the causal/thematic coherence factor, and one item from the causal coherence scale cross-loaded on the temporal coherence factor (0.36). Factor correlations ranged from 0.23 (temporal coherence with awareness) to 0.59 (causal/thematic coherence with awareness). In the four-factor solution, the causal/thematic coherence factor was split into two factors, composed of the items of the causal coherence and thematic coherence scales, respectively. The item from the causal coherence scale (“I understand how the story of my life has unfolded”) retained its cross-loading (0.33) on the temporal coherence factor. The factor correlations ranged from 0.30 (awareness with temporal coherence) to 0.72 (causal coherence with thematic coherence).

**Table 3:** EFA factor loadings and factor correlations of the ANIQ items for the three- and four-factor models.

Item	Scale	Three factors			Four factors			
		F1	F2	F3	F1	F2	F3	F4
1	Awareness	**0.31**	0.03	**0.51**	−0.02	0.00	0.28	**0.59**
2	Awareness	0.01	0.08	**0.77**	0.03	0.07	−0.07	**0.77**
3	Awareness	0.04	0.02	**0.71**	−0.02	0.08	−0.04	**0.71**
4	Awareness	−0.01	0.06	**0.80**	0.00	−0.05	−0.02	**0.88**
5	Awareness	0.11	0.07	**0.59**	0.03	0.03	0.06	**0.62**
6	Temporal coherence	0.03	**0.87**	0.04	**0.86**	−0.02	0.05	0.04
7	Temporal coherence	−0.05	**0.95**	0.03	**0.94**	−0.02	−0.02	0.02
8	Temporal coherence	−0.01	**0.93**	0.00	**0.93**	0.00	0.00	−0.01
9	Temporal coherence	−0.02	**0.97**	0.00	**0.98**	0.05	−0.05	−0.04
10	Temporal coherence	0.13	**0.78**	0.03	**0.78**	0.03	0.10	0.03
11	Causal coherence	**0.62**	**0.36**	−0.08	**0.33**	−0.02	**0.63**	0.02
12	Causal coherence	**0.80**	0.14	−0.06	0.09	−0.01	**0.81**	0.07
13	Causal coherence	**0.79**	−0.05	0.02	−0.09	0.10	**0.71**	0.10
14	Causal coherence	**0.94**	0.01	−0.15	−0.05	0.04	**0.92**	−0.04
15	Causal coherence	**0.82**	0.07	−0.08	0.03	0.09	**0.74**	0.00
16	Thematic coherence	**0.68**	−0.01	0.24	0.03	**0.77**	0.11	0.02
17	Thematic coherence	**0.68**	−0.08	0.28	−0.02	**0.98**	−0.04	−0.03
18	Thematic coherence	**0.66**	−0.02	0.29	0.03	**0.92**	−0.02	0.01
19	Thematic coherence	**0.61**	0.05	0.17	0.06	**0.46**	0.25	0.07
20	Thematic coherence	**0.64**	−0.04	0.31	0.00	**0.80**	0.04	0.08
Factor intercorrelations		F1	F2	F3	F1	F2	F3	F4
F1	–			–				
F2	0.51	–		0.40	–			
F3	0.59	0.23	–	0.52	0.72	–		
F4				0.30	0.69	0.54	–	

Factor loadings ≥0.30 in bold.

### Factor structure of the NISE

The results of the CFA of the NISE items showed an acceptable model fit according to two indices (*CFI* = 0.91, *RMSEA* = 0.10), while the *TLI* was just below the threshold for acceptability (0.89). In the EFA, the EKC indicated that extracting four factors was appropriate. The results from the parallel analysis showed that the fourth observed eigenvalue was marginally smaller than the fourth mean eigenvalue from random data (the first four observed eigenvalues were 4.194, 3.352, 0.926, and 0.275 vs. 0.556, 0.396, 0.332, and 0.278 from random data), suggesting extracting three factors. As with the ANIQ items, two EFAs were conducted with three and four factors using oblimin rotation. The factor loadings and factor correlations of the two solutions are displayed in Table 4. In the three-factor solution, the first factor was defined by the items of the positive themes scale and high negative loadings of the items of the narrative disturbances scale. The items of the autobiographical reasoning and desire for structure scales loaded highly on Factors 2 and 3, respectively. There were no substantial secondary loadings (i.e., ≥0.30). Factors 2 and 3 were moderately correlated (0.48), while the other two factor correlations were ≤ 0.08. When four factors were extracted, the positive themes and narrative disturbances scales emerged as separate factors in addition to the autobiographical reasoning and desire for structure factors. The correlations between the autobiographical reasoning and the desire for structure factors (0.47) and the positive themes and narrative disturbances factors (−0.63) were medium and large, respectively.

**Table 4:** EFA factor loadings and factor correlations of the NISE items for the three- and four-factor models.

Item	Scale	Three factors			Four factors			
		F1	F2	F3	F1	F2	F3	F4
1	Autobiographical reasoning	0.10	**0.65**	0.07	**0.66**	0.03	0.07	−0.08
2	Autobiographical reasoning	−0.05	**0.79**	−0.01	**0.78**	0.02	0.00	0.08
3	Autobiographical reasoning	−0.17	**0.75**	0.05	**0.74**	−0.04	0.06	0.14
4	Autobiographical reasoning	0.02	**0.84**	0.00	**0.87**	−0.08	0.00	−0.10
5	Autobiographical reasoning	0.26	**0.57**	−0.01	**0.56**	0.24	−0.01	−0.05
6	Desire for structure	−0.17	0.03	**0.51**	0.03	−0.13	**0.51**	0.05
7	Desire for structure	−0.04	−0.04	**0.78**	−0.02	−0.06	**0.78**	−0.02
8	Desire for structure	0.10	0.06	**0.56**	0.04	0.18	**0.56**	0.07
9	Desire for structure	0.06	0.14	**0.67**	0.13	0.11	**0.67**	0.04
10	Desire for structure	0.01	−0.04	**0.84**	−0.02	−0.02	**0.83**	−0.04
11	Positive themes	**0.52**	0.21	0.18	0.20	**0.41**	0.18	−0.16
12	Positive themes	**0.59**	0.09	0.01	0.03	**0.75**	0.00	0.09
13	Positive themes	**0.80**	−0.04	−0.02	−0.09	**0.78**	−0.03	−0.11
14	Positive themes	**0.73**	0.07	0.04	0.02	**0.74**	0.04	−0.06
15	Positive themes	**0.34**	0.01	0.13	−0.01	**0.33**	0.13	−0.05
16	Narrative disturbances	–**0.56**	0.17	−0.12	0.11	0.01	−0.12	**0.65**
17	Narrative disturbances	–**0.53**	−0.01	0.11	−0.03	−0.23	0.11	**0.35**
18	Narrative disturbances	–**0.63**	0.13	−0.01	0.08	−0.13	0.00	**0.58**
19	Narrative disturbances	–**0.67**	0.01	0.14	−0.03	−0.21	0.15	**0.54**
20	Narrative disturbances	–**0.68**	0.01	−0.01	−0.08	0.02	−0.01	**0.81**
Factor intercorrelations		F1	F2	F3	F1	F2	F3	F4
F1	–			–				
F2	0.03	–		0.08	–			
F3	0.08	0.48	–	0.47	0.10	–		
F4				0.04	−0.63	−0.03	**–**	

Factor loadings ≥0.30 in bold.

Taken together, while the CFA results for both inventories were somewhat ambiguous, the EFAs supported the proposed four-factor structures of the ANIQ and the NISE items.

### Criterion validity

The correlations between the ANIQ and NISE scales are displayed in Table 5. As hypothesized, large correlations (*rs* ≥ 0.50) between NISE autobiographical reasoning and ANIQ awareness and ANIQ thematic coherence and statistically significant negative associations between NISE narrative disturbances and all ANIQ scales, which were small to moderate in size, were found. In addition, NISE desire for structure had correlations above 0.50 with ANIQ causal coherence and ANIQ thematic coherence.

**Table 5:** Correlations between the ANIQ, NISE, LPFS-BF 2.0, and PID5BF+M scales.

Scale	ANIQ				NISE				LPFS-BF 2.0			PID5BF+M					
	Awareness	Temporal coherence	Causal coherence	Thematic coherence	Autobiographical reasoning	Desire forstructure	Positive themes	Narrativedisturbances	Total score	Self	Interpersonal	Negative affectivity	Detachment	Antagonism	Disinhibition	Psychoticism	Anankastia
ANIQ																	
Awareness	–																
Temporal coherence	0.32^***^	–															
Causal coherence	0.57^***^	0.56^***^	–														
Thematic coherence	0.68^***^	0.44^***^	0.75^***^	–													
NISE																	
Autobiographical reasoning	0.56^***^	0.16^**^	0.43^***^	0.50^***^	–												
Desire for structure	0.45^***^	0.46^***^	0.52^***^	0.52^***^	0.45^***^	–											
Positive themes	0.37^***^	0.22^***^	0.42^***^	0.35^***^	0.17^**^	0.19^***^	–										
Narrative disturbances	−0.15^**^	−0.17^**^	−0.29^***^	−0.16^**^	0.03	0.00	−0.60^***^	–									
LPFS-BF 2.0																	
Total score	−0.01	−0.22^***^	−0.20^***^	−0.09	0.12^*^	−0.03	−0.45^***^	0.65^***^	–								
Self-functioning	0.03	−0.16^**^	−0.14^*^	−0.03	0.17^**^	0.01	−0.40^***^	0.61^***^	0.93^***^	–							
Interpersonal functioning	−0.07	−0.26^***^	−0.24^***^	−0.15^**^	0.02	−0.08	−0.42^***^	0.54^***^	0.85^***^	0.59^***^	–						
PID5BF+M																	
Negative affectivity	0.17^**^	−0.05	0.02	0.15^**^	0.29^***^	0.18^**^	−0.31^***^	0.44^***^	0.65^***^	0.68^***^	0.44^***^	–					
Detachment	−0.10	−0.18^**^	−0.24^***^	−0.15^**^	−0.08	−0.10	−0.47^***^	0.50^***^	0.60^***^	0.49^***^	0.60^***^	0.33^***^	–				
Antagonism	0.01	−0.17^**^	−0.06	−0.05	0.02	−0.05	−0.17^**^	0.29^***^	0.39^***^	0.30^***^	0.41^***^	0.29^***^	0.41^***^	–			
Disinhibition	−0.03	−0.21^***^	−0.09	0.00	0.08	−0.05	−0.16^**^	0.39^***^	0.54^***^	0.49^***^	0.47^***^	0.44^***^	0.36^***^	0.49^***^	–		
Psychoticism	0.00	−0.12^*^	−0.06	0.02	0.17^**^	0.07	−0.30^***^	0.44^***^	0.53^***^	0.47^***^	0.48^***^	0.48^***^	0.46^***^	0.43^***^	0.56^***^	–	
Anankastia	0.10	−0.07	−0.04	0.06	0.21^***^	0.13^*^	−0.25^***^	0.36^***^	0.52^***^	0.51^***^	0.39^***^	0.63^***^	0.37^***^	0.36^***^	0.38^***^	0.57^***^	–

ANIQ, Awareness of Narrative Identity Questionnaire; LPFS-BF 2.0, Level of Personality Functioning Scale-Brief Form version 2.0; NISE, Narrative Identity Self-Evaluation; PID5BF+M, Personality Inventory for DSM-5- Brief Form Plus Modified. ^***^ *p* < 0.001 ^**^ *p* < 0.01 ^*^ *p* < 0.05.

The correlations of the ANIQ and NISE scales with the LPFS-BF 2.0 and PID5BF+M scales are also presented in Table 5. Consistent with expectations, among the NISE and ANIQ scales, NISE narrative disturbances showed the strongest association with the LPFS-BF 2.0 total score (*r* = 0.65, *p* < 0.001). The correlation with the LPFS-BF 2.0 self-functioning subscale (*r* = 0.61, *p* < 0.001) was slightly higher than the correlation with the interpersonal functioning subscale (*r* = 0.54, *p* < 0.001). NISE positive themes showed moderate negative associations with the LPFS-BF 2.0 subscales, while the NISE autobiographical reasoning and desire for structure scales were weakly or non-significantly related to the LPFS-BF 2.0 scales. ANIQ temporal coherence and ANIQ causal coherence were significantly negatively correlated with the LPFS-BF 2.0 scales, with small to medium effect sizes. Furthermore, the hypothesis of weaker associations of NISE narrative disturbances with the PID5BF+M scales than with the LPFS-BF 2.0 scales was supported. The correlations ranged from *r* = 0.29 (antagonism) to *r* = 0.50 (detachment) (all *ps* < 0.001). Similarly, NISE positive themes was significantly related to all PID5BF+M scales, with correlation coefficients ranging from *r* = −0.16 (*p* < 0.01) for disinhibition to *r* = −0.47 for detachment (*p* < 0.001). In addition, several small to medium-sized correlations of the remaining NISE scales and the ANIQ scales with the PID5BF+M scales were found, the highest being between NISE autobiographical reasoning and PID5BF+M negative affectivity (*r* = 0.29, *p* < 0.001) and between ANIQ causal coherence and PID5BF+M detachment (*r* = −0.24, *p* < 0.001). Sensitivity analyses using Spearman correlations for the associations involving NISE autobiographical reasoning and LPFS-BF 2.0 interpersonal functioning showed that the correlations with NISE autobiographical reasoning were slightly changed (most were reduced in the range 0.02–0.04, the largest reduction was 0.07 with ANIQ awareness). Similarly, the correlations remained largely unchanged for LPFS-BF 2.0 interpersonal functioning (most changes were in the range +/−0.02, the largest change was an increase of 0.05 with PID5BF+M antagonism) when using Spearman's rho.

## Discussion

Narrative identity is increasingly recognized as an important domain of personality that is relevant to the understanding of psychopathology, especially personality disorders, a field that is currently undergoing a fusion between personality and personality pathology (Lind et al., 2022). The ANIQ and the NISE were developed to aid the assessment of narrative identity through self-report. While the ANIQ focuses on the metacognitive awareness of one's life story and its temporal, causal, and thematic coherence, the NISE is broader, encompassing all three main domains of narrative identity (autobiographical reasoning, motivational and affective themes, and structural features) as well as disrupted narrative identity. This study evaluated the psychometric properties of the Norwegian versions of the ANIQ and the NISE by examining the instruments' internal consistency, structural validity, and criterion validity in relation to personality functioning and maladaptive personality traits.

As hypothesized and consistent with the original version, the Norwegian ANIQ showed high internal consistency. Overall, its proposed four-factor structure was supported, although the results of the CFA and EFA were less clear than in previous reports (Balzen et al., 2024; Dierdorp et al., 2023; Hallford and Mellor, 2017). One index (RMSEA) suggested an unsatisfactory fit of the data with the hypothesized model in the CFA, and one extraction method indicated three rather than four factors in the EFA, with the causal and thematic coherence items of the ANIQ merging into one factor. Nevertheless, the four-factor solution for the ANIQ items aligned with the inventory's proposed internal structure, with only one ANIQ item showing a cross-loading. One reason for the ambiguous factor-analytic findings can be that the constructs assessed by the ANIQ are theoretically and empirically interrelated (Hallford and Mellor, 2017). For example, the three different forms of coherence assessed with the ANIQ are theorized to build on one another (Habermas and Bluck, 2000). These inherent relationships in the ANIQ can increase the risk of difficulties distinguishing scales in factor analyses, as was observed for the Farsi version of the ANIQ (Mazaheri-Nejad-Fard et al., 2025). From a statistical viewpoint, the precision of methods to determine the number of factors deteriorates as factor correlations increase (Auerswald and Moshagen, 2019). The pattern and magnitude of the intercorrelations among the ANIQ scales in the Norwegian sample were highly similar to those observed in previous investigations (Dierdorp et al., 2023; Hallford and Mellor, 2017; Sevim and Otrar, 2021), including the increasing correlations of the awareness scale with the temporal, causal, and thematic coherence scales, respectively, and the strong association between ANIQ causal coherence and ANIQ thematic coherence.

Similar to the Norwegian ANIQ, results for the Norwegian NISE showed high internal consistency of the scales and overall support for its proposed four-factor structure. The pattern of intercorrelations between the NISE scales aligned with the results of the inventory development study (Lind et al., 2025). In the current study, the association between NISE narrative disturbances and NISE positive themes was stronger (−0.60) than the highest correlation between the scales in the previous investigation (−0.49 in sample 2 in Lind et al., 2025), which may explain why the two scales merged in the three-factor solution of the EFA. However, despite the substantial overlap, the size of the correlation coefficient does not indicate a lack of discriminant validity or redundancy of the two NISE scales. As for the ANIQ, intercorrelations among the NISE subscales are expected because, in the underlying model on which the inventory is based (McLean et al., 2020), the domains of narrative identity are interrelated. Similarly, NISE narrative disturbances covers maladaptive variants of the three other normal-range narrative identity scales, making it conceptually related to those scales. For both the ANIQ and the NISE, methodological factors, such as solely positively keyed items and item grouping, may increase the within-inventory correlations. However, including reverse-keyed items in an inventory can reduce the internal consistency of a scale, and evidence on the benefits of randomizing item order is mixed (Stefana et al., 2025).

Regarding the relationships between the two self-report measures of narrative identity, the study's hypotheses were supported by the results and largely replicated the findings by Lind et al. (2025). First, the ANIQ and NISE scales were statistically significantly correlated, suggesting that the two instruments assess related constructs. Second, the awareness and the thematic coherence scales of the ANIQ showed strong associations with the autobiographical reasoning scale of the NISE, which corroborates the notion that these features of narrative identity can be best subsumed under the autobiographical reasoning domain (McLean et al., 2020). However, non-hypothesized large correlations of NISE desire for structure with ANIQ causal coherence and ANIQ thematic coherence were also observed. Thus, in the current sample, the desire for structure in the life story was tied to experiencing meaningful connections between life events and identifying common themes. It therefore appears that the NISE desire for structure scale captures elements of both the autobiographical reasoning and the structure domain in the McLean et al. (2020) framework. Finally, as expected, since the ANIQ scales cover adaptive features of narrative identity, they were negatively associated with NISE narrative disturbances.

Given the relevance of narrative identity to the new models of personality disorders in the DSM-5 AMPD and the ICD-11, the instruments' relationships with external criteria were investigated by examining their associations with measures of personality functioning and maladaptive trait domains. For the ANIQ, the correlations that were statistically significant were generally negative and in the small to medium range. The temporal coherence scale had the most significant associations across the personality functioning and the DSM-5 AMPD/ICD-11 trait scales, suggesting that self-perceived difficulties with temporally ordering life events may be a general feature of personality pathology. Trait detachment was significantly negatively related to the three forms of narrative coherence assessed by the ANIQ. In the DSM-5 AMPD/ICD-11, detachment refers to the tendency to avoid socioemotional experience [American Psychiatric Association, 2013; World Health Organization (WHO), 2019/2021]. On the other hand, narrative coherence is linked to socioemotional reciprocity and positive social relationships (Foldager et al., 2024; Waters and Fivush, 2015). The observed negative relationship between self-reported detachment and narrative coherence may therefore reflect shared dysfunctional socioemotional processes. It should be noted, however, that several studies have not found associations between rater-coded narrative coherence and personality functioning and pathological personality traits (e.g., Baaijens et al., 2024; Dimitrova and Simms, 2022; See et al., 2023). Thus, the correlations between self-perceived narrative coherence and personality pathology observed in the current study may reflect shared method variance. However, it is also conceivable that self-report and coder-rated measures of narrative identity variables assess overlapping but distinct constructs, e.g., qualities of narrative identity that the person is aware of vs. more implicit features (Panattoni and McLean, 2018). Notably, except for trait negative affectivity, self-reported metacognitive awareness of one's life story was unrelated to personality pathology. The correlations of ANIQ awareness and ANIQ thematic coherence, i.e., the two scales that assess autobiographical reasoning, with negative affectivity were positive. Since negative affectivity is associated with a tendency to ruminate (Lysiak, 2019), these findings may indicate a relationship between negative affectivity and ruminating about one's life.

Turning to the NISE results, a positive association between negative affectivity and self-reported autobiographical reasoning was again found using this inventory. In addition, NISE autobiographical reasoning showed small to medium positive correlations with impaired personality functioning, especially self-functioning, and with the trait domains psychoticism and anankastia. As hypothesized, the NISE narrative disturbances scale, which was developed to assess maladaptive features of narrative identity as compared to the NISE autobiographical reasoning, desire for structure, and positive themes scales and the ANIQ scales, which cover normal-range and adaptive narrative identity features, showed the strongest associations with impaired self- and interpersonal functioning and pathological personality traits. This finding supports the scale's conceptualization as assessing disrupted narrative identity and its relevance to personality pathology. Moreover, as predicted, self-reported narrative disturbances were more strongly related to impaired personality functioning than to pathological personality traits. Lind et al. (2022) demonstrated that narrative identity is related to different elements of personality functioning in the DSM-5 AMPD and the ICD-11. Thus, the study findings support the construct validity of the NISE narrative disturbances scale. However, medium to large associations were also observed between the NISE narrative disturbances scale and all six pathological personality trait domains. This may partly be due to the maladaptive content of the scales used. It may also point to connections between the domains of narrative identity and personality traits (McAdams et al., 2004). In addition to NISE narrative disturbances, NISE positive themes was significantly (negatively) associated with impaired personality functioning and all six pathological personality traits. Previous investigations have reported a relationship only between negative affectivity and a negative content of life narratives in addition to impaired personality functioning (Baaijens et al., 2024; de Moor et al., 2022). In these studies, the valence of narratives was assessed by raters rather than through self-report as in the current investigation. As with the associations between self-perceived narrative coherence and personality pathology, shared method variance may have inflated the correlations between the scales in the present sample. Nevertheless, the results suggest that personality pathology is associated with a negative content of narrative identity, which can be addressed in clinical interventions (Çili et al., 2017).

Some limitations of the current study should be considered when interpreting the results. First, traditional measurement of narrative identity (i.e., rater-based coding of narratives) was not included in the study, preventing an examination of the convergence of the Norwegian versions of the ANIQ and the NISE with the established assessment of narrative identity. Second, since the participants were recruited in an academic environment, the sample is likely not representative of the Norwegian population with an overrepresentation of highly educated individuals. In addition, the sample was predominantly female, which further limits the generalizability of the results. Notably, women scored higher on NISE autobiographical reasoning than men. However, this gender difference also appears in content-coded narrative identity (Fivush, 2011), indicating that the self-report measure functions similarly to the traditional way of assessing narrative identity. Moreover, most participants reported no or only mild impairment in personality functioning, suggesting an underrepresentation of clinical participants. This range restriction of personality pathology may have attenuated the observed correlations between the narrative identity and the personality pathology scales. An investigation into the psychometric properties of the ANIQ and the NISE in more diverse Norwegian samples, including community and clinical samples, is desirable. Third, the present study examined the criterion validity of the Norwegian ANIQ and NISE only with respect to personality pathology. Investigating associations with other domains of personality, such as normal-range traits (e.g., the Big Five) and characteristic adaptations (McAdams, 2013), would be a valuable next step in the psychometric evaluation of these instruments. Finally, the cross-sectional design of the study prevents drawing causal conclusions about the relationships between the study measures.

In conclusion, the study's findings suggest that the internal consistency of the original ANIQ and NISE scales is preserved in the Norwegian versions of the instruments. Overall support for the proposed internal structure of the instruments was found, although the evidence was somewhat mixed in regard to the results from the CFAs. The study also highlighted the meaningful relationships between self-reported narrative identity and personality pathology.

## Data Availability

The raw data supporting the conclusions of this article will be made available by the authors, without undue reservation.
