# Supplementary material for: Self-reported narrative identity in Norway: psychometric evaluation of the Awareness of Narrative Identity Questionnaire and the Narrative Identity Self-Evaluation scale with focus on personality pathology
Source: Front Psychol. 2026 Jun 15;17:1834608. doi: 10.3389/fpsyg.2026.1834608 (PMC13312161; doi:10.3389/fpsyg.2026.1834608)
Supplement: Supplementary file 1 [file Supplementary_File_1.pdf]

## Supplementary material

**Bevissthet om Narrativ Identitet Spørreskjema (ANIQ)**
(David John Hallford og David Mellor, 2017)

Alle har minner om opplevelser de har hatt i løpet av livet sitt. Noen ganger kan minnene brukes til å skape historier om livene våre. Følgende påstander handler om hvordan du kan bruke minnene dine til å forstå hvilken slags person du har vært, hvem du er, og hvem du forventer å bli.

Du kan svare på påstandene under på en skala fra 0 (helt uenig) til 10 (helt enig), der en *høyere* score indikerer *sterkere* enighet. Prøv å besvare spørsmålene bredt og i forhold til hvordan du generelt bruker dine personlige minner, i stedet for å prøve å relatere dem til spesifikke omstendigheter eller opplevelser.

1. Minnene mine er som historier som hjelper meg å forstå min identitet.
2. Jeg bruker historiene mine om livet mitt for å finne ut hva slags person jeg er.
3. Opplevelsene fra fortiden min lager historien om hvem jeg er.
4. Hvordan jeg forstår meg selv er forankret i minner fra livet mitt.
5. Når jeg tenker tilbake på livet mitt, merker jeg at det er en historie som forteller meg hvem jeg er.
6. Jeg kan plassere hendelsene i livet mitt i rekkefølge etter når de skjedde.
7. Det er enkelt for meg å vite rekkefølgen der hendelsene i livet mitt skjedde.
8. Når jeg tenker tilbake på opplevelser jeg har hatt, vet jeg når de skjedde i livet mitt.
9. Jeg har god bevissthet om rekkefølgen når hendelser og opplevelser i livet mitt skjedde.
10. Når jeg tenker på opplevelser i fortiden, er det enkelt å huske hva som skjedde før og etter dem.
11. Jeg forstår hvordan historien om livet mitt har utspilt seg.
12. Jeg forstår hvordan opplevelsene i livet mitt er knyttet til hverandre.
13. Ting som har skjedd i løpet av livet mitt er meningsfullt knyttet sammen.
14. Jeg er klar over hvordan hendelser i livet mitt er koblet sammen.
15. Jeg kan forstå hvordan opplevelser i livet mitt har skjedd, med én ting som fører til en annen.
16. Når jeg tenker eller snakker om opplevelser i fortiden min, kan jeg se temaer om hva slags person jeg er.
17. Jeg kan oppfatte felles temaer om hvem jeg er i minner fra livet mitt.
18. Jeg legger merke til temaer i de personlige minnene fra livet mitt som relaterer seg til hva slags person jeg er.
19. Når jeg husker hendelser og opplevelser gjennom livet, kan jeg se klare mønstre i måten jeg tenker, føler og handler på.
20. I mine personlige minner, finnes det tydelige temaer som knytter seg til hvem jeg er.

**Narrativ Identitet Selv-Evaluering (NISE)**
(Majse Lind, Raffles Cowan, Jonathan Adler, Dan McAdams, 2025)

Instruksjoner: Noen mennesker tenker på hvem de er, og hvordan de har utviklet seg over tid, i form av personlige historier. Disse historiene kan omfatte den overordnede livshistorien eller kortere historier fra livet. Du kan føle at dette stemmer for hvordan du ser deg selv, eller kanskje ikke. Vennligst reflekter over hvordan du tenker på deg selv som en person når du svarer på følgende spørsmål (items). Det finnes ingen riktige eller gale svar. Hvis det hjelper, kan du ta et øyeblikk nå for å tenke på hvordan du engasjerer deg med historier fra ditt liv (hvis du gjør det i det hele tatt), og deretter svare på følgende spørsmål:

1. Å tenke på livshistorien min hjelper meg med å forstå hvem jeg er som person.
2. Jeg er nysgjerrig på å forstå hvordan hendelser i livshistorien min har forandret meg som person.
3. Jeg tenker mye på forbindelser mellom ulike opplevelser jeg har hatt (f.eks., hvordan én opplevelse forårsaket en senere opplevelse).
4. Jeg har lært mye og fått innsikt ved å tenke på livshistorien min.
5. Ettersom tiden går, legger jeg merke til at jeg i økende grad lærer og vokser på det jeg har opplevd.
6. Jeg vil ha fakta riktig når jeg tenker på historier fra livet mitt.
7. I de personlige historiene mine synes jeg det er viktig å vite hvor og når ting har skjedd.
8. Historier fra livet mitt har vanligvis en tydelig begynnelse, midtpunkt og slutt.
9. Det betyr noe for meg å ha en sammenhengende livshistorie.
10. Jeg vil få riktig tidslinje for livshistorien min, så langt som mulig.
11. Når jeg ser tilbake på livshistorien min, gjenkjenner jeg en følelse av tilhørighet med andre mennesker.
12. De fleste dårlige ting som har skjedd i livshistorien min har løst seg til slutt på en positiv måte.
13. Totalt sett vil jeg betrakte livshistorien min som mer positiv enn negativ.
14. Historien om livet mitt er en veldig optimistisk historie.
15. I livshistorien min har mine egne personlige beslutninger i stor grad vært den drivende kraften, i motsetning til ytre omstendigheter.
16. Når jeg tenker på livshistorien min, ender jeg opp med å føle meg forvirret om hvem jeg er som person.
17. Jeg har hatt ingen kontroll over hva som har skjedd i historien om livet mitt.
18. I livshistorien min ser jeg en hovedperson som ikke har vært i stand til å elske og bli elsket av andre.
19. I livshistorien min kan jeg legge merke til et mønster der gode hendelser, selv de mest lovende, til slutt ble dårlige.
20. Livshistorien min føles ut som et puslespill, der brikkene ikke passer sammen.
