# Supplementary material for: Self-reported narrative identity in Norway: psychometric evaluation of the Awareness of Narrative Identity Questionnaire and the Narrative Identity Self-Evaluation scale with focus on personality pathology
Source: Front Psychol. 2026 Jun 15;17:1834608. doi: 10.3389/fpsyg.2026.1834608 (PMC13312161; doi:10.3389/fpsyg.2026.1834608)
Supplement: Supplementary file 2 [file Supplementary_File_2.pdf]

Narrative Identity Self-Evaluation (NISE)

Majse Lind, Raffles Cowan, Jonathan Adler, Dan McAdams

Instructions: Some people think about who they are and how they have developed over time in the form of personal stories. These stories may encompass the overall life story or shorter stories from one's life. You may feel like this is true to how you see yourself, or not really. Please reflect on how you think about yourself as a person when answering the following questions. There are no right or wrong answers. If it helps, take a moment now to think about how you engage with stories from your life (if at all), and then answer the following questions.

No.	Item	Response						
		Strongly disagree	Disagree	Somewh at disagree	Neutral	Some what agree	Agree	Strongly agree
1	Thinking about my life story helps me know who I am as a person							
2	I am curious to understand how events in my life story have changed me as a person							
3	I think a lot about connections between different experiences I have had (e.g., how one experience caused a later experience)							
4	I have learned lessons and gained insights from thinking about my life story							
5	As time passes, I notice that I increasingly learn and grow from my experiences							
6	I want to get the facts right when I think about stories from my life							
7	In my personal stories, I think it's important to know where and when things have happened							
8	Stories from my life usually have a clear beginning, middle, and end							

9	It matters to me to have a coherent life story							
10	I want to get the timeline of my life story correct, as much as possible							
11	Looking back on my life story, I recognize a sense of belongingness with other people							
12	Most bad things that have happened in my life story have eventually resolved in a positive way							
13	Overall, I would consider my life story to be more positive than negative							
14	The story of my life is a highly optimistic one							
15	In my life story, my own personal decisions have largely been the driving force, rather than external circumstances							
16	When I think about my life story, I end up feeling confused about who I am as a person							
17	I have had no control over what has happened in the story of my life							
18	In my life story, I see a protagonist unable to love and be loved by others							
19	In my life story, I notice a pattern of how good events, even the most promising ones, eventually turned bad							
20	My life story feels like a puzzle and the pieces don't fit together							

Subscales:

Autobiographical reasoning: 1-5, Narrative structure: 6-10, positive themes: 11-15, narrative identity disturbance: 16-20
